# Associations of Cytomegalovirus Infection With All-Cause and Cardiovascular Mortality in Multiple Observational Cohort Studies of Older Adults

**DOI:** 10.1093/infdis/jiaa480

**Published:** 2020-09-10

**Authors:** Sijia Chen, Graham Pawelec, Stella Trompet, David Goldeck, Laust H Mortensen, P Eline Slagboom, Kaare Christensen, Jacobijn Gussekloo, Patricia Kearney, Brendan M Buckley, Ian Ford, J Wouter Jukema, Rudi G J Westendorp, Andrea B Maier

**Affiliations:** 1 Department of Gerontology and Geriatrics, Leiden University Medical Centre, Leiden, the Netherlands; 2 Department of Experimental Immunology, Academic Medical Center, Amsterdam, the Netherlands; 3 Department of Immunology, University of Tübingen, Tübingen, Germany; 4 Health Sciences North Research Institute, Sudbury, Ontario, Canada; 5 Fairfax Centre, Kidlington, United Kingdom; 6 Methods and Analysis, Statistics Denmark, Copenhagen, Denmark; 7 Section of Molecular Epidemiology, Leiden University Medical Centre, Leiden, the Netherlands; 8 Danish Aging Research Center, University of Southern Denmark, Odense, Denmark; 9 Department of Public Health and Primary Care, Leiden University Medical Centre, Leiden, the Netherlands; 10 Department of Epidemiology and Public Health, University College Cork, Cork, Ireland; 11 Department of Pharmacology and Therapeutics, University College Cork, Cork, Ireland; 12 Robertson Center for Biostatistics, University of Glasgow, United Kingdom; 13 Department of Cardiology, Leiden University Medical Center, Leiden, the Netherlands; 14 Leyden Academy on Vitality and Ageing, Leiden, the Netherlands; 15 Department of Public Health, University of Copenhagen, Copenhagen, Denmark; 16 Department of Medicine and Aged Care, @AgeMelbourne, The Royal Melbourne Hospital, University of Melbourne, Melbourne, Australia; 17 Department of Human Movement Sciences, @AgeAmsterdam, Amsterdam Movement Sciences, Vrije University, Amsterdam, the Netherlands

**Keywords:** Herpesviridae, cytomegalovirus, seroepidemiologic studies, immunoglobulin G, mortality, cardiovascular, aged

## Abstract

**Background:**

Whether latent cytomegalovirus (CMV) infection in older adults has any substantial health consequences is unclear. Here, we sought associations between CMV-seropositivity and IgG titer with all-cause and cardiovascular mortality in 5 longitudinal cohorts.

**Methods:**

Leiden Longevity Study, Prospective Study of Pravastatin in the Elderly at Risk, Longitudinal Study of Aging Danish Twins, and Leiden 85-plus Study were assessed at median (2.8–11.4 years) follow-up . Cox regression and random effects meta-analysis were used to estimate mortality risk dependent on CMV serostatus and/or IgG antibody titer, in quartiles after adjusting for confounders.

**Results:**

CMV-seropositivity was seen in 47%–79% of 10 122 white community-dwelling adults aged 59–93 years. Of these, 3519 had died on follow-up (579 from cardiovascular disease). CMV seropositivity was not associated with all-cause (hazard ratio [HR], 1.05; 95% confidence interval [CI], .97–1.14) or cardiovascular mortality (HR, 0.97; 95% CI, .83–1.13). Subjects in the highest CMV IgG quartile group had increased all-cause mortality relative to CMV-seronegatives (HR, 1.16; 95% CI, 1.04–1.29) but this association lost significance after adjustment for confounders (HR, 1.13; 95% CI, .99–1.29). The lack of increased mortality risk was confirmed in subanalyses.

**Conclusions:**

CMV infection is not associated with all-cause or cardiovascular mortality in white community-dwelling older adults.

## Teaser

CMV infection is not associated with all-cause or cardiovascular mortality in Caucasian community-dwelling older adults.

**(See the Editorial Commentary by Nikolich-Žugich, on pages 181–3.)**

Human cytomegalovirus (CMV) is a herpes virus that is carried by 70%–100% of older adults worldwide, depending on age and socioeconomic factors [1]. The immune system normally controls but can never eliminate the virus, which establishes a latent infection with subclinical intermittent reactivations [2]. The presence of anti-CMV immunoglobulin G (IgG) in the blood is evidence of previous CMV infection. Severe clinical manifestations of CMV infection are common in immunocompromised [3] and critically ill patients [4] but the clinical relevance of latent CMV infection remains to be established [5]. In recent years, CMV seropositivity has been increasingly linked to cardiovascular diseases [6, 7], diabetes [8, 9], and other age-related comorbidities [8, 10, 11]. In line with this, high CMV IgG titers have also been associated with cardiovascular [12] and other diseases [13, 14]. In addition, CMV seropositivity and CMV IgG titer have been linked to all-cause mortality and at times cardiovascular mortality, but not with any consistency. In 3 large cohorts, CMV seropositivity was reported to be associated with higher all-cause mortality risk [15–17] and to cardiovascular mortality risk [16, 18], but CMV seropositivity was not reported to be associated with mortality risk in some other cohorts [8, 19, 20]. There were also disparities in the associations reported between high CMV IgG titers and mortality. Some cohorts showed increased all-cause mortality risk for subjects within the highest CMV IgG quartile [14, 15, 21, 22], whereas others revealed no association with all-cause mortality [8, 23], nor with cardiovascular mortality risk [15–17, 24].

Overall, the differences in parameters of CMV infection (CMV seropositivity or high IgG quartiles) and reference groups assessed (CMV-negative status or lowest IgG quartile) and confounders adjusted for, make cross-cohort comparisons complex. Furthermore, the association between CMV infection and mortality may be confounded by socioeconomic status [1] and it remains unclear if the potentially increased mortality risk is due to increased cardiovascular deaths [25] or noncardiovascular events.

We aimed to clarify the potential association between CMV infection and mortality and included multiple cohorts of older community-dwelling adults over a broad age range, each with large numbers of covariates available to enable adjustments for confounders. We were also able to define 2 reference groups, that is a CMV-seronegative group and the lowest quartile of CMV IgG antibodies group. First, we set out to systematically assess associations between both parameters of previous CMV infection (CMV serostatus and CMV IgG titers divided into quartiles) with all-cause mortality in 5 observational cohorts of community-dwelling older adults. Next, we examined the mortality risk separately for cardiovascular and noncardiovascular mortality and subsequently pooled all data in meta-analyses.

## METHODS

### Study Design

Cohorts were selected based on availability of long-term mortality data, data of confounding variables, and either CMV serology data or biomaterial available to measure CMV status. The following 5 observational cohorts were included: (1) the Leiden Longevity Study F2 (LLS F2), is a cohort studying familial longevity, consisting of 2429 middle-aged offspring of nonagenarian siblings, and their partners (data collection 2002–2011) [26]; (2) the Prospective Study of Pravastatin in the Elderly at Risk (PROSPER) is an at-risk cohort for cardiovascular disease, including 5639 subjects recruited for having an increased risk of cardiovascular disease (data collection 1997–2016) [27]; (3) the Longitudinal Study of Ageing in Danish Twins (LSADT) is a cohort to elucidate the cause of variation in survival, health, diseases, loss of abilities, and cognitive function, consisting of 604 Danish twins aged 70 years and older (data collection 1997–2010) [28]; (4) the Leiden 85-plus Study is a cohort representing the oldest old population with 549 subjects aged 85 years at inclusion (data collection 1997–2011) [29]; and (5) the Leiden Longevity Study F1 (LLS F1) is a cohort enriched for familiar longevity consisting of 901 nonagenarians (data collection 2002–2011) [26]. Details of individual cohorts have been described elsewhere [26–29] and ethical approval was obtained for all individual cohorts.

### CMV Serostatus and CMV IgG Antibody Titer

CMV serostatus was determined by enzyme-linked immunosorbent assay (ELISA) using the CMV-IgG-ELISA PKS assay (Medac) in LLS F2 [30]. In the PROSPER study, Leiden 85-plus Study, and in LLS F1, CMV IgG ETI-CYTOK-G PLUS (DiaSorin) was used and the anti-CMV-IgG kit (Dade Behring, Marburg) was used in LSADT [9, 30]. All experiments were performed according to the manufacturer’s instructions on blinded samples. CMV IgG titers are displayed in international units (IU) [30] or in antibody units/mL [9].

### Mortality

For LLS F2, Leiden 85-plus Study, and LLS F1 individuals were followed for occurrence of mortality until 31 December 2011. For PROSPER, follow-up was until 31 December 2016. We obtained dates of deaths from the Dutch civic registry and specific data on the causes of death from Statistics Netherlands. For LSADT, data up until 31 December 2010 were obtained from the Danish Civil Registry. For all cohorts, cause of death was coded using the international classification of diseases ICD-10. I00-I99 from ICD-10 was classified as death from cardiovascular causes. Remaining events were classified as noncardiovascular mortality. In total there were 3519 events from all-cause mortality, including 579 instances of cardiovascular mortality.

#### LLS F2

Follow-up period was median 8.7 years (interquartile range [IQR], 7.8–9.4): n = 113 of n = 2429 subjects died, n = 8 from cardiovascular causes.

#### PROSPER

Follow-up period was median 6.8 years (IQR, 6.5–7.1) for all-cause mortality: n = 1705 of all subjects (n = 5639) died. For cardiovascular mortality the follow-up period was 3.25 years (IQR, 3.0–3.5): n = 50 subjects died from cardiovascular causes.

#### LSADT

Follow-up period was median 11.4 years (IQR, 7.2–16.0): n = 427 of all subjects (n = 604) died; n = 138 died from cardiovascular causes.

#### Leiden 85-Plus Study

Follow-up period was median 5.3 years (IQR, 2.5–8.3): n = 494 of all subjects (n = 549) died; n = 180 died from cardiovascular causes.

#### LLS F1

Follow-up period was median 2.8 years (IQR, 1.3–4.7): n = 780 of all subjects (n = 901) died and n = 203 died from cardiovascular causes.

### Covariates

Variables considered as confounders were those that have a relationship with CMV serostatus and/or CMV IgG antibody titer, as well as a causal relationship with (disease-specific) mortality: age, sex [31], body mass index (BMI) [32], education [33], smoking status, number of comorbidities [6–14], number of medications, and C-reactive protein (CRP) [25]. For PROSPER, additional potential confounders were included: country of residence and statin use. BMI was calculated using self-reported or measured height and weight at baseline. Education was dichotomized into high and low education; for the LLS F2, LSADT, and the Leiden 85-plus Study individuals who went to elementary school or had less than 8 years of schooling were assigned to the low education group. In PROSPER, education was defined as the age at which subjects left school, and was analyzed as a continuous variable. Smoking status was dichotomized into never or ever smoked. Information on comorbidities was obtained from subjects’ general practitioners and the number of medications was obtained from the subjects’ pharmacies. To determine the number of comorbidities, we summed the presence of cardiovascular comorbidities (hypertension, myocardial infarction, angina pectoris, stroke, and intermittent claudication), diabetes, chronic obstructive pulmonary disease (COPD), cancer, and arthritis. Plasma CRP was assayed by a high-sensitivity ELISA in LLS F2 (Roche) and in LSADT (Dade Behring), by a particle-enhanced immunoturbidimetric assay in PROSPER (Roche), and by the Hitachi 747 system in the Leiden 85-plus Study. For LLS F1, there was no information available on these covariates except for sex and age.

### Statistical Analyses

Means with standard deviation (SD) or median values with IQR are presented. CMV IgG titer was divided into quartiles for each cohort separately. In LLS F2, the upper cutoff value for CMV IgG measurement was given as >15 IU, therefore CMV IgG titer was dichotomized.

Cox regressions with age as underlying time were performed for each cohort separately to estimate the mortality risk of CMV serostatus and CMV IgG antibody titer (in quartiles) for all-cause mortality and cardiovascular mortality separately.

First, CMV-seropositive subjects were compared to CMV-seronegative subjects. Second, for analyzing CMV IgG antibody titers, CMV-seronegative subjects were used as reference groups. Third, the highest CMV IgG quartile was compared to the lowest CMV IgG quartile.

The following adjustments for confounders were applied: model 1 was adjusted for age and sex (for PROSPER, also country and statin use); model 2 included model 1 and education, BMI, smoking status, number of comorbidities, and medication; finally, model 3 included model 2 and CRP. Due to heterogeneity of the cohorts, as measured with the I-square statistic, random effects model meta-analysis was applied to obtain pooled estimates from hazard ratios (HR) from 5 cohorts. Analyses were performed in SPSS 20, meta-analysis were performed in StataSE 30, and confirmed in RevMan 5, in which the forest-plots were made. *P* values < .05 were considered statistically significant.

## RESULTS

Characteristics of the 10 122 subjects from 5 cohorts are presented in Table 1. Mean age ranged from 59 to 93.4 years. Prevalence of CMV seropositivity was 47% in the LLS F2, 70% in the PROSPER, 76% in the LSADT, 79% in the Leiden 85-plus Study, and 59% in the LLS F1.

**Table 1.** Characteristics of Individuals from 5 Observational Cohort Studies

	LLS F2	PROSPER	LSADT	Leiden 85-plus	LLS F1
Characteristics	(n = 2429)	(n = 5639)	(n = 604)	(n = 549)	(n = 901)
Age, y, mean (SD)	59.2 (6.8)	75.3 (3.4)	78.2 (4.4)	85 (0)	93.4 (2.6)
Women, n (%)	1325 (54.5)	3000 (51.7)	455 (66.0)	368 (67.0)	553 (61.4)
Low education, n (%)^a^	92 (4.6)	15.1 (2.0)	468 (68.1)	357 (65.4)	NA
BMI, mean (SD)	25.4 (3.6)	26.8 (4.2)	24.2 (3.8)	27.2 (4.5)	NA
Smoking ever, n (%)	1279 (63.8)	3835 (66.1)	426 (62.1)	261 (47.8)	NA
Hypertension, n (%)	486 (20.4)	3592 (61.9)	184 (26.7)	215 (40.2)	NA
Myocardial infarction, n (%)	58 (2.4)	776 (13.4)	42 (6.1)	125 (23.4)	NA
Angina pectoris, n (%)	1 (0.0)	649 (11.2)	43 (6.3)	260 (48.6)	NA
Stroke, n (%)	63 (2.6)	649 (11.2)	39 (5.7)	49 (9.2)	NA
Intermittent claudication, n (%)	3 (0.1)	390 (6.7)	118 (17.1)	32 (6.0)	NA
Diabetes, n (%)	105 (4.4)	623 (10.7)	40 (5.8)	84 (15.7)	NA
COPD, n (%)	73 (3.0)	NA	65 (9.4)	63 (11.8)	NA
Cancer, n (%)	172 (7.1)	NA	85 (12.3)	95 (17.8)	NA
Arthritis, n (%)	23 (1.0)	NA	242 (35.1)	171 (32.1)	NA
Number of comorbidities, median (IQR)	0 (0–1)	1 (1–2)	1 (0–2)	1 (1–3)	NA
Number of medications, median (IQR)	NA	3 (2–5)	2 (1–4)	3 (1–5)	NA
CRP, median (IQR)	1.3 (0.7–2.7)	3.1 (1.6–6.2)	1.6 (0.9–4.2)	4.0 (1.0–8.0)	NA
CMV seropositivity, n (%)	1136 (46.8)	3972 (70.4)	461 (76.3)	434 (79.1)	536 (59.5)

Abbreviations: BMI, body mass index; CMV, cytomegalovirus; COPD, chronic obstructive pulmonary disease; CRP, C-reactive protein; F1, siblings of long-living participants; F2, offspring; IQR, interquartile range; LLS, Leiden Longevity Study; LSADT, Longitudinal Study of Ageing in Danish Twins; NA, not available; PROSPER, Prospective Study of Pravastatin in the Elderly at Risk.

^a^LLS F2, Leiden 85 Plus and LSADT: elementary school only or less than 8 years education, n (%). PROSPER: age left school, years, mean (SD).

**Figure 1.**
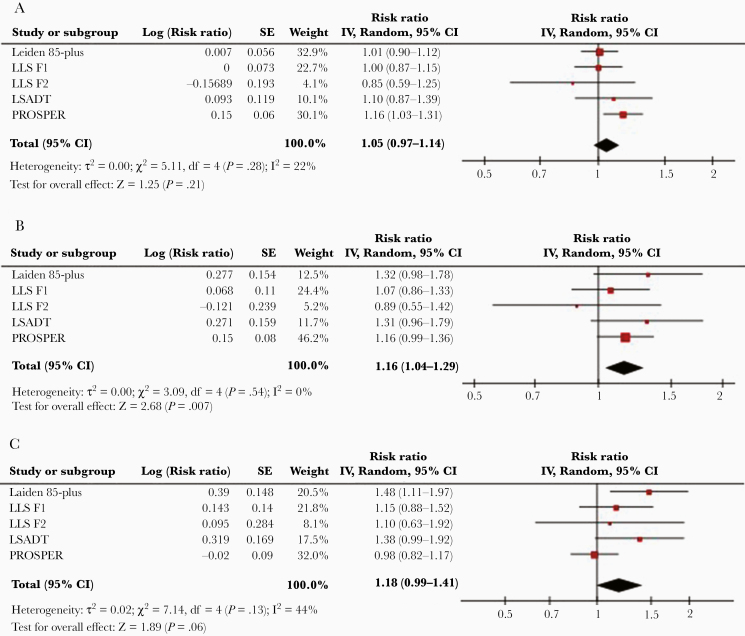
Meta-analysis of all-cause mortality and CMV infection in 5 cohorts. *A*, Meta-analysis of the association between all-cause mortality risk and CMV seropositivity in comparison to CMV-seronegative individuals. *B*, Comparison of individuals in the highest antibody quartile (IgG4) to CMV-negative individuals. *C*, Comparison of individuals in the highest antibody quartile (IgG4) to ones in the lowest antibody quartile (IgG1). Abbreviations: CI, confidence interval; CMV, cytomegalovirus; F1, siblings of long-living participants; F2, offspring; IgG, immunoglobulin G; IV, inverse variance; LLS, Leiden Longevity Study; LSADT, Longitudinal Study of Ageing in Danish Twins; PROSPER, Prospective Study of Pravastatin in the Elderly at Risk; SE, standard error.

**Figure 2.**
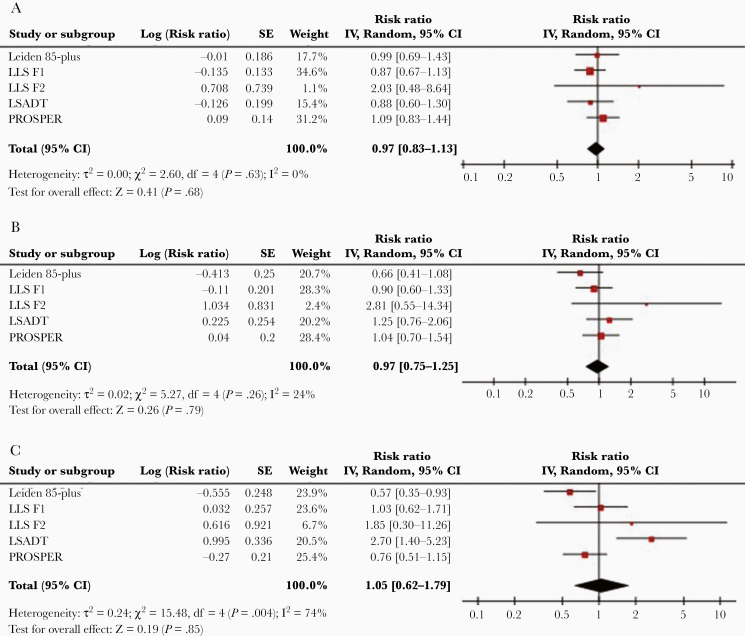
Meta-analysis of cardiovascular mortality and CMV infection in 5 cohorts. *A*, Meta-analysis of the association between cardiovascular mortality risk and CMV seropositivity in comparison to CMV-seronegative individuals. *B*, Comparison of individuals in the highest antibody quartile (IgG4) to CMV-negative individuals. *C*, Comparison of individuals in the highest antibody quartile (IgG4) to ones in the lowest antibody quartile (IgG1). Abbreviations: CI, confidence interval; CMV, cytomegalovirus; F1, siblings of long-living participants; F2, offspring; IgG, immunoglobulin G; IV, inverse variance; LLS, Leiden Longevity Study; LSADT, Longitudinal Study of Ageing in Danish Twins; PROSPER, Prospective Study of Pravastatin in the Elderly at Risk; SE, standard error.

First, CMV serostatus was found not to be significantly associated with all-cause mortality (model 1: HR, 1.05; 95% CI, .97–1.14; Figure 1A and Table 2, pooled estimates of all models). Of the 5 cohorts included in the analyses, only PROSPER showed a significant association between CMV positivity and all-cause mortality (model 1: HR, 1.16; 95% CI, 1.03–1.31; Supplementary Table 1). The statistical significance of this association was lost after further adjustment (model 2: HR, 1.09; 95% CI, .98–1.22; Supplementary Table 1). Further analyses for cardiovascular mortality (pooled estimate, model 1: HR, 0.97; 95% CI, .83–1.13; Figure 2A and Table 2, pooled estimates of all models) and noncardiovascular mortality (pooled estimate, model 1: HR, 1.09; 95% CI, .97–1.23; Supplementary Figure 1A and Supplementary Table 3, pooled estimates of all models) confirmed that there were no significant associations of CMV serostatus with mortality. CMV serostatus was not associated with cardiovascular mortality (Supplementary Table 1) or noncardiovascular mortality (Supplementary Table 4) in any of the cohorts.

**Table 2.** Pooled Estimates of the Associations Between CMV Seropositivity or CMV IgG Antibody Quartiles with All-Cause and Cardiovascular Mortality, Compared to CMV-Seronegative Status

Cytomegalovirus	All-Cause Mortality, HR (95% CI)			Cardiovascular Mortality, HR (95% CI)		
	Model 1^b^	Model 2^c^	Model 3^c^	Model 1^b^	Model 2^c^	Model 3^c^
Seronegativity^a^	1	1	1	1	1	1
Seropositivity all	1.05 (.97–1.14)	1.07 (.99–1.16)	1.07 (.99–1.16)	0.97 (.83–1.13)	1.00 (.82–1.21)	1.00 (.82–1.2)
IgG antibody quartile 1	0.98 (.84–1.15)	0.97 (.80–1.18)	0.99 (.82–1.20)	0.96 (.66–1.38)	0.94 (.56–1.59)	0.93 (.55–1.58)
IgG antibody quartile 2	1.07 (.96–1.19)	1.01 (.88–1.15)	1.02 (.90–1.16)	0.94 (.76–1.16)	0.85 (.65–1.11)	0.83 (.61–1.12)
IgG antibody quartile 3	1.08 (.92–1.27)	1.13 (.99–1.29)	1.13 (.99–1.29)	1.03 (.76–1.40)	1.17 (.90–1.51)	1.15 (.89–1.49)
IgG antibody quartile 4	1.16 (1.04–1.29)	1.13 (.99–1.29)	1.13 (.99–1.29)	0.97 (.75–1.25)	0.96 (.72–1.26)	0.96 (.72–1.26)

Cox regression analyses within individual cohorts (Supplementary Table 1) were performed in 3 models:

Model 1: adjustment for age and sex (for PROSPER, also country and statin use);

Model 2: adjustment for model 1 plus body mass index, education, smoking status, number of comorbidities, and medications;

Model 3: adjustment for model 2 plus log transformed C-reactive protein.

Abbreviations: CMV, cytomegalovirus; CI, confidence interval; HR, hazard ratio; IgG, immunoglobulin G; PROSPER, Prospective Study of Pravastatin in the Elderly at Risk.

^a^CMV-seronegative individuals were the reference group.

^b^All cohorts included.

^c^All cohorts except for Leiden Longevity Study (LLS) F1 and F2.

Second, when compared to CMV-seronegative subjects, the all-cause mortality risk for subjects in the highest CMV IgG quartile was significantly higher (pooled estimate, model 1: HR, 1.16; 95% CI, 1.04–1.29; Figure 1B). This association was overall nonsignificant for all other CMV IgG quartiles (Table 2). None of the studied cohorts showed a significant association of the highest CMV IgG quartile with all-cause mortality compared to the CMV-seronegative subjects (Supplementary Table 1). The overall association lost statistical significance after adjustment for confounders in model 2 (pooled estimates HR, 1.13; 95% CI, .99–1.29; Table 2). Cardiovascular (Figure 2B and Table 2) and noncardiovascular mortality (Supplementary Figure 1B and Supplementary Table 3) were also not associated with the highest CMV IgG quartile when compared to CMV-seronegative subjects (pooled estimate HR, 0.97; 95% CI, .75–1.25 and HR, 1.06; 95% CI, .86–1.30, respectively). Cardiovascular mortality and noncardiovascular mortality were not associated with the highest IgG quartile when compared to CMV-seronegative subjects in any of the studied cohorts (Supplementary Table 1 and Supplementary Table 4).

Third, when compared to subjects within the lowest CMV IgG quartile, subjects in the highest CMV IgG quartile did not suffer increased all-cause mortality (pooled estimate, model 1: HR, 1.18; 95% CI, .98–1.42; Figure 1C and Table 3). Of the 5 cohorts studied, only subjects of the Leiden 85-plus Study within the highest IgG quartile had a significantly higher mortality compared to subjects in the lowest IgG quartile (model 1: HR, 1.48; 95% CI, 1.11–1.97; Supplementary Table 2). This association remained significant after further adjustment (model 3: HR, 1.39; 95% CI, 1.04–1.87; Supplementary Table 2). For cardiovascular mortality, the pooled estimate was not statistically significant for subjects in the highest IgG quartile when compared to the lowest IgG quartile (model 1: HR, 1.05; 95% CI, .62–1.79; Figure 2C). Of the 5 cohorts, the highest IgG quartile was associated with a statistically significantly higher cardiovascular mortality risk in the LSADT (model 3: HR, 2.45; 95% CI, 1.26–4.78; Supplementary Table 2) and significantly lower cardiovascular mortality risk in the Leiden 85-plus Study (model 3: HR, 0.60; 95% CI, .37–.97; Supplementary Table 2). When compared to subjects within the lowest CMV IgG quartile, subjects in the highest CMV IgG quartile did not have an increased noncardiovascular mortality (model 1: HR, 0.96; 95% CI, .80–1.15; Supplementary Figure 1C and Supplementary Table 5).

**Table 3.** Pooled Estimates of the Associations Between Higher CMV IgG Antibody Quartiles With All-Cause and Cardiovascular Mortality, Compared to the Lowest CMV IgG Quartile

Cytomegalovirus	All-Cause Mortality, HR (95% CI)			Cardiovascular Mortality, HR (95% CI)		
	Model 1^b^	Model 2^c^	Model 3^c^	Model 1^b^	Model 2^c^	Model 3^c^
IgG antibody quartile 1^a^	1	1	1	1	1	1
IgG antibody quartile 2	1.01 (.78–1.30)	0.99 (.87–1.14)	0.98 (.85–1.21)	0.99 (.68–1.44)	0.88 (.60–1.29)	0.87 (.60–1.26)
IgG antibody quartile 3	1.06 (.95–1.20)	1.08 (.95–1.23)	1.09 (.90–1.31)	1.09 (.70–1.68)	1.24 (.70–2.20)	1.24 (.69–2.21)
IgG antibody quartile 4	1.18 (.98–1.42)	1.18 (.91–1.54)	1.15 (.89–1.49)	1.05 (.62–1.79)	1.01 (.48–2.09)	0.99 (.48–2.06)

Cox regression analyses within individual cohorts (Supplementary Table 1) were performed in 3 models:

Model 1: adjustment for age and sex (for PROSPER, also country and statin use);

Model 2: adjustment for model 1 plus body mass index, education, smoking status, number of comorbidities, and medications;

Model 3: adjustment for model 2 plus log transformed C-reactive protein.

Abbreviations: CMV, cytomegalovirus; CI, confidence interval; HR, hazard ratio; IgG, immunoglobulin G.

^a^CMV IgG antibody quartile 1 was the reference group.

^b^All cohorts included.

^c^All cohorts except for Leiden Longevity Study (LLS) F1 and F2.

## DISCUSSION

In 5 cohorts of community-dwelling older adults, neither CMV seropositivity nor high CMV IgG titer quartiles were significantly associated with increased all-cause mortality risk. This is the first large-scale analysis providing systematic assessments of CMV status and CMV IgG antibody quantiles using different reference groups (both CMV-negative status as well as the lowest CMV IgG quartile). We have included both reference groups because previous studies on CMV IgG antibody titers and mortality have either used the lowest CMV IgG quartile [21] or negative CMV status as control [14, 22] and reported inconsistent results. We are also the first to report additional analyses for cardiovascular mortality and noncardiovascular mortality. These analyses confirmed the lack of increased mortality risk for CMV-seropositive subjects and for subjects in high IgG quartiles.

The hypothesis that latent CMV infection could accelerate mortality was based on little causal evidence. In fact, the hypothetical pathways were mainly based on cross-sectional associations and were instrumental in our selection of potential covariate adjustments, in addition to established confounders such as socioeconomic status [1, 21] and age. First, latent CMV infection is hypothesized to increase systemic inflammation by subclinical reactivations [34, 35] but studies have failed to demonstrate this [21, 25, 36, 37]. Because CMV seropositivity may be associated with higher CRP values [17], which is in turn associated with comorbidities and independently predicts adverse outcomes [38], we added CRP to our confounder adjustments. The influence of CRP in the final adjustment model (model 3) did not change the overall results, shown by only small changes in effect sizes between model 2 including education, BMI, smoking status, number of comorbidities, and medication, and the final model including CRP. A second hypothesis regarding the mechanism by which CMV may increase progression of disease and thereby mortality is based on associations of CMV infection with the prevalence of cardiovascular disease [39], diabetes [9] or cancer [40]. Because there is no clear causal evidence for these associations, increased CMV seropositivity and/or titers may be the consequence of disease burden and not the reason for their onset. We therefore also considered comorbidities in our confounder adjustments. Third, CMV seropositivity is associated with hallmarks of immunosenescence and thereby proposed to diminish response to influenza vaccination [41]. It is, however, unclear whether immunosenescence phenotypes are necessarily detrimental [42] and data on influenza vaccine responsiveness do not consistently support this hypothesis [43].

Previous cohorts for which a lack of association between CMV serostatus and mortality were reported were much smaller compared to the present study [8, 19, 20, 44]. In contrast, studies demonstrating a positive association between CMV seropositivity and all-cause mortality included 3 larger cohorts [15–17]. An association between CMV seropositivity and cardiovascular mortality has been reported in a recent meta-analysis (relative risk, 1.30; 95% CI, 1.03–1.66) [45]; however, 2 studies with the highest effect size only minimally adjusted for possible confounders (age and sex). The differences between our study and previous cohorts may be explained by the additional adjustments for health parameters employed here, especially for systemic inflammation. In support of this, within PROSPER, which included subjects of similar in age to the ERSC Healthy Aging Study, we observed that the association between CMV seropositivity and cardiovascular mortality was present in the first model, but did not remain significant after adjustment for additional confounders in model 2 and in model 3, which included CRP. In the ERSC Healthy Aging Study, systemic inflammatory factors were not included as covariates [16].

We also failed to find an association between CMV IgG antibody titer and mortality. This negative finding is in line with reports from several cohorts regarding all-cause [8, 23] or cardiovascular mortality risk [15–17, 24]. In previous studies that did report a positive association, no adjustments for socioeconomic factors [22], inflammatory markers [16, 21], and comorbidities [14] had been made. In comparison to these cohorts, our population had a lower prevalence of CMV across age groups [14, 21], and potentially there were differences in socioeconomic status, which is associated with the acquisition of CMV and other health consequences [1, 21].

The strengths of our study are the inclusion of older community-dwelling adults over a broad age range, the large numbers of subjects included, and the broad availability of data allowing adjustments for multiple confounders. We were also able to define 2 reference groups, that is a CMV-seronegative group and the lowest quartile of CMV IgG antibodies group. This makes it possible to compare our analyses with previous published data. Moreover, we were able to perform subanalyses for cardiovascular and noncardiovascular mortality. Limitations include lack of investigating the effect of CMV in other ethnicities because most subjects in our cohorts were white; our confounder adjustments for socioeconomic status could have been more robust by including income levels in addition to education [46]; and when analyzing cardiovascular and noncardiovascular mortality, potential errors could have been introduced due to inaccurate certification of death in older people, especially when multiple comorbidities are present in the elderly. Furthermore, information on immunodeficiency virus infection and immunosuppressant agents was not available for our cohorts, which might have influenced our results. However, the risk profile of participants in our cohorts is rather low.

## CONCLUSION

CMV infection is not associated with all-cause and cardiovascular mortality in multiple different cohorts of white community-dwelling older adults. The impact of CMV serostatus and IgG antibody titers on aging and mortality should be cautiously revisited.

## Supplementary Data

Supplementary materials are available at *The Journal of Infectious Diseases* online. Consisting of data provided by the authors to benefit the reader, the posted materials are not copyedited and are the sole responsibility of the authors, so questions or comments should be addressed to the corresponding author.

jiaa480_suppl_Supplementary_Table_S1

jiaa480_suppl_Supplementary_Table_S2

jiaa480_suppl_Supplementary_Table_S3

jiaa480_suppl_Supplementary_Table_S4

jiaa480_suppl_Supplementary_Table_S5

jiaa480_suppl_Supplementary_Figure_1

## Notes

***Acknowledgments.*** We thank Dr Karel Beenakker and Yotam Raz for feedback on the draft of the manuscript. The authors acknowledge participation of all study subjects, without whom this project would not have been possible.

***Author contributions.*** A. B. M., S. C., G. P., and R. G. J. W. developed the concept of this study. L. H. M., P. E. S., K. C., J. G., R. G. J. W., and A. B. M. collected the data for the study. The first draft of the manuscript was written by S. C. and revised by G. P., S. T., D. G., L. H. M., P. E. S., K. C., J. G., P. K., B. M. B., I. F., J. W. J., R. G. J. W., and A. B. M. S. C. and A. B. M. had full access to all of the data in the study and take responsibility for the integrity of the data and the accuracy of the data analysis.

***Disclaimer.*** The funders had no role in study design, data collection and analysis, decision to publish, or preparation of the manuscript.

***Financial support.*** This work was supported by the European Commission (grant numbers FP6 036894 LifeSpan, FP7 259679 IDEAL, FP7 HEALTH-2007-2.4.5-10 Myoage, FP7 HEALTH-F2-2009–223004); SenterNovem Innovation Oriented Research Program on Genomics (grant numbers IGE01014 and IGE5007); the Centre for Medical Systems Biology, and the Netherlands Genomics Initiative/Netherlands Organization for Scientific Research (grant numbers 05040202 and 050-060-810); the Ministry of Health, Welfare and Sports; and the US National Institutes of Health, National Institute on Aging (grant number P01 AG08761).

***Potential conflicts of interest.*** All authors: No reported conflicts of interest. All authors have submitted the ICMJE Form for Disclosure of Potential Conflicts of Interest. Conflicts that the editors consider relevant to the content of the manuscript have been disclosed.
