# Supplementary material for: Associations of Cytomegalovirus Infection With All-Cause and Cardiovascular Mortality in Multiple Observational Cohort Studies of Older Adults
Source: J Infect Dis. 2020 Sep 10;223(2):238–46. doi: 10.1093/infdis/jiaa480 (PMC7857154; doi:10.1093/infdis/jiaa480)
Supplement: jiaa480_suppl_Supplementary_Table_S1 [file jiaa480_suppl_supplementary_table_s1.docx]

**Supplementary table S1: Associations between CMV seropositivity and CMV IgG antibody quartiles with all-cause and cardiovascular mortality, compared to CMV seronegativity within individual cohorts.**

|  | | All-cause mortality, HR (95% CI) | | |  | Cardiovascular mortality, HR (95% CI) | | |
| --- | --- | --- | --- | --- | --- | --- | --- | --- |
| *Cytomegalovirus* | | Model 1 | Model 2 | Model 3 |  | Model 1 | Model 2 | Model 3 |
| Seronegativity^a^: | | 1 | 1 | 1 |  | 1 | 1 | 1 |
| Seropositivity all: | | |  |  |  |  |  |  |
|  | LLS F2 | 0.86 (0.59; 1.25) | n.a. | n.a. |  | 2.03 (0.48; 8.64) | n.a | n.a. |
|  | PROSPER | 1.16 (1.03; 1.31) | 1.09 (0.98; 1.22) | 1.10 (0.98; 1.23) |  | 1.09 (0.83; 1.44) | 1.02 (0.78; 1.34) | 1.03 (0.78; 1.35) |
|  | LSADT | 1.10 (0.87; 1.39) | 1.10 (0.87; 1,40) | 1.09 (0.86; 1.38) |  | 0.88 (0.60; 1.30) | 0.90 (0.61; 1.34) | 0.90 (0.61; 1.34) |
|  | Leiden 85-plus | 1.01 (0.90; 1.12) | 1.04 (0.93; 1.17) | 1.03 (0.92; 1.16) |  | 0.99 (0.69; 1.43) | 1.04 (0.71; 1.52) | 1.03 (0.70; 1.51) |
|  | LLS F1 | 1.00 (0.87; 1.15) | n.a | n.a |  | 0.87 (0.67; 1.13) | n.a. | n.a. |
|  | Pooled estimate estimate | 1.05 (0.97; 1.14) | 1.07 (0.99; 1.16) | 1.07 (0.99; 1.15) |  | 0.97 (0.83; 1.13) | 1.00 (0.82; 1.21) | 1.00 (0.82; 1.2) |
|  |  |  |  |  |  |  |  |  |
|  | IgG antibody quartile 1: | |  |  |  |  |  |  |
|  | LLS F2^b^ | 0.83 (0.52; 1.32) | n.a. | n.a. |  | 1.58 (0.26; 9.62) | n.a. | n.a. |
|  | PROSPER | 1.18 (1.02; 1.37)  ) | 1.11 (0.95; 1.29) | 1.13 (0.97; 1.31) |  | 1.29 (0.92; 1.82) | 1.17 (0.83; 1.66) | 1.20 (0.84; 1.69) |
|  | LSADT | 0.94 (0.69; 1.28) | 0.92 (0.67; 1.26) | 0.90 (0.65; 1.24) |  | 0.47 (0.25; 0.89) | 0.48 (0.25; 0.91) | 0.47 (0.25; 0.90) |
|  | Leiden 85-plus | 0.90 (0.68; 1.20) | 0.81 (0.60; 1.10) | 0.85 (0.62; 1.15) |  | 1.23 (0.76; 1.99) | 1.28 (0.77; 2.06) | 1.23 (0.74; 2.06) |
|  | LLS F1 | 0.89 (0.72; 1.11) | n.a. | n.a. |  | 0.89 (0.72; 1.11) | n.a. | n.a. |
|  | Pooled estimate | 0.98 (0.84; 1.14) | 0.97 (0.80; 1.19) | 0.99 (0.82; 1.20) |  | 0.97 (0.71; 1.33) | 0.94 (0.56; 1.59) | 0.93 (0.55; 1.58) |
|  |  | |  |  |  |  |  |  |
|  | IgG antibody quartile 2: | |  |  |  |  |  |  |
|  | LLS F2^b^ | n.a | n.a. | n.a. |  | n.a | n.a. | n.a. |
|  | PROSPER | 1.10 (0.94; 1.28) | 1.04 (0.89; 1.22) | 1.05 (0.90; 1.23) |  | 0.66 (0.39; 1.11) | 0.70 (0.41; 1.19) | 0.73 (0.43; 1.25) |
|  | LSADT | 1.02 (0.76; 1.38) | 1.10 (0.81; 1.49) | 1.10 (0.80; 1.48) |  | 0.87 (0.58; 1.28) | 0.82 (0.55; 1.22) | 0.83 (0.56; 1.24) |
|  | Leiden 85-plus | 0.87 (0.66; 1.16) | 0.83 (0.61; 1.12) | 0.84 (0.62; 1.14) |  | 0.97 (0.62; 1.53) | 1.01 (0.63; 1.63) | 1.00 (0.62; 1.61) |
|  | LLS F1 | 1.18 (0.95; 1.46) | n.a. | n.a. |  | 1.15 (0.79; 1.66) | n.a. | n.a. |
|  | Pooled estimate | 1.07 (0.96; 1.19) | 1.01 (0.88;1.15) | 1.02 (0.90; 1.16) |  | 0.94 (0.76; 1.16) | 0.85 (0.65; 1.11) | 0.83 (0.61; 1.12) |
|  |  | |  |  |  |  |  |  |
|  | IgG antibody quartile 3: | |  |  |  |  |  |  |
|  | LLS F2^b^ | n.a | n.a. | n.a. |  | n.a | n.a. | n.a. |
|  | PROSPER | 1.25 (1.07; 1.46) | 1.17 (1.00; 1.36) | 1.17 (1.00; 1.36) |  | 1.27 (0.89; 1.83) | 1.19 (0.82; 1.72) | 1.18 (0.81; 1.70) |
|  | LSADT | 1.18 (0.87; 1.60) | 1.24 (0.91; 1.69) | 1.21 (0.88; 1.65) |  | 1.22 (0.74; 1.99) | 1.33 (0.80; 2.21) | 1.31 (0.80; 2.18) |
|  | Leiden 85-plus | 0.93 (0.70; 1.22) | 0.94 (0.69; 1.28) | 0.96 (0.70; 1.31) |  | 1.10 (0.69; 1.75) | 0.98 (0.59; 1.64) | 0.96 (0.57; 1.61) |
|  | LLS F1 | 0.95 (0.76; 1.19) | n.a. | n.a. |  | 0.65 (0.41; 1.02) | n.a. | n.a. |
|  | Pooled estimate | 1.08 (0.92; 1.28) | 1.14 (1.00; 1.29) | 1.14 (1.00; 1.29) |  | 1.03 (0.76; 1.39) | 1.17 (0.90;1.51) | 1.15 (0.89; 1.49) |
|  | | |  |  |  |  |  |  |
| IgG antibody quartile 4: | | |  |  |  |  |  |  |
|  | LLS F2^b^ | 0.89 (0.55; 1.42) | n.a. | n.a. |  | 2.81 (0.55; 14.3) | n.a. | n.a. |
|  | PROSPER | 1.16 (0.99; 1.36) | 1.11 (0.94; 1.30)  0 | 1.10 (0.94; 1.29) |  | 1.04 (0.70; 1.54) | 1.00 (0.67; 1.49) | 0.99 (0.66; 1.47) |
|  | LSADT | 1.31 (0.96; 1,79) | 1.17 (0.85; 1.61) | 1.21 (0.88; 1.66) |  | 1.25 (0.76; 2.06) | 1.19 (0.72; 1.96) | 1.20 (0.72; 1.99) |
|  | Leiden 85-plus | 1.32 (0.98; 1.78) | 1.21 (0.87; 1.67) | 1.20 (0.86; 1.66) |  | 0.66 (0.41; 1.08) | 0.69 (0.41; 1.18) | 0.70 (0.41; 1.19) |
|  | LLS F1 | 1.07 (0.86; 1.33) | n.a. | n.a. |  | 0.90 (0.60; 1.33) | n.a | n.a |
|  | Pooled estimate | 1.16 (1.04; 1.29) | 1.13 (0.99; 1.29) | 1.13 (0.99; 1.29) |  | 0.97 (0.75; 1.25) | 0.96 (0.72; 1.26) | 0.96 (0.72; 1.26) |

HR: hazard ratio. CI: confidence interval. IgG: Immunoglobulin.

^a^ CMV seronegative individuals were the reference group.

^b^ For Leiden Longevity Study (LLS) F2, IgG antibody level was dichotomized instead of divided in quartiles due to rounded off values.

Cox regression analyses within individual cohorts were performed in 3 models:

Model 1: adjustment for age and sex (for PROSPER, also country and statin use).

Model 2: adjustment for model 1 plus Body Mass Index, education, smoking status, numbers of comorbidities and of medication.

Model 3: adjusted for model 2 plus log transformed C-reactive protein.
