# Supplementary material for: Associations of Cytomegalovirus Infection With All-Cause and Cardiovascular Mortality in Multiple Observational Cohort Studies of Older Adults
Source: J Infect Dis. 2020 Sep 10;223(2):238–46. doi: 10.1093/infdis/jiaa480 (PMC7857154; doi:10.1093/infdis/jiaa480)
Supplement: jiaa480_suppl_Supplementary_Table_S2 [file jiaa480_suppl_supplementary_table_s2.docx]

**Supplementary table S2: Associations between higher CMV IgG antibody quartiles with all-cause and cardiovascular mortality, compared to the lowest CMV IgG quartile within individual cohorts.**

|  | All-cause mortality, HR (95% CI) | | | |  | Cardiovascular mortality, HR (95% CI) | | |
| --- | --- | --- | --- | --- | --- | --- | --- | --- |
| *Cytomegalovirus* | Model 1 | | Model 2 | Model 3 |  | Model 1 | Model 2 | Model 3 |
| IgG antibody quartile 1^a^: 1 | | 1 | 1 | 1 |  | 1 | 1 | 1 |
| IgG antibody quartile 2: | | |  |  |  |  |  |  |
| LLS F2^b^ | n.a. | | n.a. | n.a |  | n.a. | n.a | n.a |
| PROSPER | 0.93 (0.79; 1.25) | | 0.96 (0.81; 1.16) | 0.95 (0.80; 1.17) |  | 0.67 (0.44; 1.09) | 0.71 (0.47; 1.02) | 0.69 (0.46; 1.03) |
| LSADT | 1.12 (0.82; 1.55) | | 1.16 (0.84; 1.61) | 1.16 (0.83; 1.60) |  | 1.46 (0.73; 2.94) | 1.52 (0.75; 3.11) | 1.47 (0.72; 3.00) |
| Leiden 85-plus | 0.95 (0.72; 1.27) | | 0.96 (0.72; 1.28) | 0.93 (0.69; 1.23) |  | 0.82 (0.52; 1.31) | 0.83 (0.52; 1.32) | 0.84 (0.52; 1.34) |
| LLS F1 | 1.29 (0.99; 1.69) | | n.a. | n.a. |  | 1.40 (0.86; 2.27) | n.a. | n.a. |
| Pooled estimate | 1.01 (0.78; 1.30) | | 0.99 (0.87; 1.14) | 0.98 (0.85; 1.21) |  | 0.99 (0.68; 1.44) | 0.88 (0.60; 1.29) | 0.87 (0.60; 1.26) |
|  |  | |  |  |  |  |  |  |
| IgG antibody quartile 3: | | |  |  |  |  |  |  |
| LLS F2^b^ | n.a | | n.a. | n.a |  | n.a | n.a | n.a |
| PROSPER | 1.05 (0.89; 1.16) | | 1.05 (0.89; 1.10)  ) | 1.03 (0.87; 1.10) |  | 0.95 (0.65; 1.18) | 0.97 (0.66; 1.11) | 0.95 (0.64; 1.13) |
| LSADT | 1.22 (0.88; 1.68) | | 1.25 (0.90; 1.73) | 1.25 (0.90; 1.73) |  | 2.54 (1.33; 4.88) | 2.60 (1.34; 5.03) | 2.63 (1.35; 5.10) |
| Leiden 85-plus | 1.04 (0.79; 1.38) | | 1.07 (0.80; 1.44) | 1.05 (0.78; 1.41) |  | 0.88 (0.54; 1.42) | 0.88 (0.53; 1.44) | 0.89 (0.54; 1.46) |
| LLS F1 | 1.04 (0.79; 1.38) | | n.a. | n.a. |  | 0.78 (0.45; 1.35) | n.a. | n.a. |
| Pooled estimate | 1.06 (0.95; 1.20) | | 1.08 (0.95; 1.23) | 1.09 (0.90; 1.31) |  | 1.09 (0.70; 1.68) | 1.24 (0.70; 2.20) | 1.24 (0.69; 2.21) |
|  |  | |  |  |  |  |  |  |
| IgG antibody quartile 4: | | |  |  |  |  |  |  |
| LLS F2^b^ | 1.10 (0.63; 1.92) | | n.a. | n.a |  | 1.85 (0.30; 11.26) | n.a | n.a |
| PROSPER | 0.98 (0.82; 1.17) | | 0.99 (0.84; 1.09) | 0.97 (0.82; 1.10) |  | 0.76 (0.51; 1.15) | 0.78 (0.52; 1.04) | 0.75 (0.50; 1.05) |
| LSADT | 1.38 (0.99; 1.92) | | 1.24 (0.89; 1.74) | 1.25 (0.89; 1.75) |  | 2.70 (1.40; 5.23) | 2.47 (1.27; 4.82) | 2.45 (1.26; 4.78) |
| Leiden 85-plus | 1.48 (1.11; 1.97) | | 1.46 (1.09; 1.96) | 1.39 (1.04; 1.87) |  | 0.57 (0.35; 0.93) | 0.59 (0.36; 0.97) | 0.60 (0.37; 0.97) |
| LLS F1 | 1.15 (0.88; 1.52) | | n.a. | n.a. |  | 1.03 (0.62; 1.71) | n.a. | n.a. |
| Pooled estimate | 1.18 (0.99; 1.41) | | 1.18 (0.91; 1.54) | 1.15 (0.89; 1.49) |  | 1.05 (0.62; 1.79) | 1.01 (0.48; 2.09) | 0.99 (0.48; 2.06) |

HR: hazard ratio. CI: confidence interval. IgG: Immunoglobulin.

^a^ CMV IgG antibody quartile 1 was the reference group.

^b^ For Leiden Longevity Study (LLS) F2, IgG antibody level was dichotomized instead of divided in quartiles due to rounded off values.

Cox regression analyses within individual cohorts were performed in 3 models:

Model 1: adjustment for age and sex (for PROSPER, also country and statin use).

Model 2: adjustment for model 1 plus Body Mass Index, education, smoking status, numbers of comorbidities and of medication.

Model 3: adjusted for model 2 plus log transformed C-reactive protein.
