# Supplementary material for: Associations of Cytomegalovirus Infection With All-Cause and Cardiovascular Mortality in Multiple Observational Cohort Studies of Older Adults
Source: J Infect Dis. 2020 Sep 10;223(2):238–46. doi: 10.1093/infdis/jiaa480 (PMC7857154; doi:10.1093/infdis/jiaa480)
Supplement: jiaa480_suppl_Supplementary_Table_S3 [file jiaa480_suppl_supplementary_table_s3.docx]

**Supplementary table S3:** Pooled estimates of the associations between CMV seropositivity and CMV IgG antibody quartiles with non-cardiovascular mortality, compared to CMV seronegative status (A) or to the lowest CMV IgG quartile (B).

**A**

| *Cytomegalovirus* | | Non-cardiovascular mortality, HR (95% CI) | | |
| --- | --- | --- | --- | --- |
|  |  | Model 1^b^ | Model 2^c^ | Model 3^c^ |
| Seronegativity^a^ | | 1 | 1 | 1 |
| Seropositivity | | 1.09 (0.97; 1.23) | 1.08 (0.91; 1.27) | 1.06 (0.90; 1.25) |
|  | IgG antibody quartile 1 | 1.14 (0.98; 1.34) | 1.15 (0.93; 1.43) | 1.13 (0.92; 1.40) |
|  | IgG antibody quartile 2 | 1.18 (1.00; 1.40) | 1.17 (0.89; 1.52) | 1.15 (0.89; 1.49) |
|  | IgG antibody quartile 3 | 1.09 (0.91; 1.29) | 1.04 (0.83; 1.31) | 1.04 (0.83; 1.31) |
|  | IgG antibody quartile 4 | 1.06 (0.86; 1.30) | 0.96 (0.76; 1.22) | 0.98 (0.77; 1.24) |

**B**

| *Cytomegalovirus* | Non-cardiovascular mortality, HR (95% CI) | | |
| --- | --- | --- | --- |
|  | Model 1^b^ | Model 2^c^ | Model 3^c^ |
| IgG antibody quartile 1^d^ | 1 | 1 | 1 |
| IgG antibody quartile 2 | 1.09 (0.91; 1.30) | 1.09 (0.89; 1.34) | 1.10 (0.89; 1.35) |
| IgG antibody quartile 3 | 0.99 (0.82; 1.18) | 0.96 (0.78; 1.19) | 0.96 (0.78; 1.19) |
| IgG antibody quartile 4 | 0.96 (0.80; 1,15) | 0.86 (0.70; 1.07) | 0.88 (0.71; 1.09) |

HR: hazard ratio. CI: confidence interval. IgG: Immunoglobulin.

^a^ CMV seronegative individuals were the reference group.

^b^ All cohorts included.

^c^ All cohorts except for Leiden Longevity Study (LLS) F1 and F2.

^d^ CMV IgG antibody quartile 1 was the reference group.

Cox regression analyses within individual cohorts (supplemental table 2) were performed in 3 models:

Model 1: adjustment for age and sex (for PROSPER, also country and statin use).

Model 2: adjustment for model 1 plus Body Mass Index, education, smoking status, number of comorbidities and medications.

Model 3: adjustment for model 2 plus log transformed C-reactive protein.
