# Supplementary material for: Associations of Cytomegalovirus Infection With All-Cause and Cardiovascular Mortality in Multiple Observational Cohort Studies of Older Adults
Source: J Infect Dis. 2020 Sep 10;223(2):238–46. doi: 10.1093/infdis/jiaa480 (PMC7857154; doi:10.1093/infdis/jiaa480)
Supplement: jiaa480_suppl_Supplementary_Table_S4 [file jiaa480_suppl_supplementary_table_s4.docx]

**Supplementary table S4: Associations between CMV seropositivity and CMV IgG antibody quartiles with non-cardiovascular mortality, compared to CMV seronegative status.**

|  | | Non-cardiovascular mortality, HR (95% CI) | | |
| --- | --- | --- | --- | --- |
| *Cytomegalovirus* | | Model 1 | Model 2 | Model 3 |
| Seronegativity^a^: | | 1 | 1 | 1 |
| Seropositivity all: | | |  |  |
|  | LLS F2 | 0.93 (0.63; 1.38) | n.a. | n.a. |
|  | PROSPER | 0.99 (0.76; 1.30) | 0.93 (0.71; 1.22) | 0.93 (0.71; 1.23) |
|  | LSADT | 1.23 (0.92; 1.65) | 1.23 (0.92; 1.66) | 1.21 (0.90; 1.63) |
|  | Leiden 85-plus | 1.03 (0.78; 1.37) | 1.11 (0.83; 1.49) | 1.08 (0.81; 1.46) |
|  | LLS F1 | 1.16 (0.95; 1.41) | n.a | n.a |
|  | Pooled estimate estimate | 1.09 (0.97; 1.23) | 1.08 (0.91; 1.27) | 1.06 (0.90; 1.25) |
|  |  |  |  |  |
|  | IgG antibody quartile 1: | |  |  |
|  | LLS F2^b^ | 0.90 (0.56; 1.47) | n.a. | n.a. |
|  | PROSPER | 1.15 (0.81; 1.63) | 1.09 (0.76; 1.55) | 1.10 (0.77; 1.57) |
|  | LSADT | 1.22 (0.85; 1.76) | 1.18 (0.81; 1.71) | 1.14 (0.79; 1.66) |
|  | Leiden 85-plus | 1.06 (0.74; 1.52) | 1.21 (0.83; 1.76) | 1.16 (0.79; 1.70) |
|  | LLS F1 | 1.25 (0.94; 1.68) | n.a. | n.a. |
|  | Pooled estimate | 1.14 (0.98; 1.34) | 1.15 (0.93; 1.43) | 1.13 (0.92; 1.40) |
|  |  | |  |  |
|  | IgG antibody quartile 2: | |  |  |
|  | LLS F2^b^ | n.a | n.a. | n.a. |
|  | PROSPER | 0.95 (0.66; 1.36) | 0.89 (0.62; 1.29) | 0.89 (0.62; 1.29) |
|  | LSADT | 1.25 (0.87; 1.80) | 1.33 (0.91; 1.92) | 1.30 (0.90; 1.88) |
|  | Leiden 85-plus | 1.28 (0.89; 1.85) | 1.35 (0.92; 2,00) | 1.34 (0.90; 1.98) |
|  | LLS F1 | 1.25 (0.94; 1.68) | n.a. | n.a. |
|  | Pooled estimate | 1.18 (1.00; 1.40) | 1.17 (0.89; 1.52) | 1.15 (0.89; 1.49) |
|  |  | |  |  |
|  | IgG antibody quartile 3: | |  |  |
|  | LLS F2^b^ | n.a | n.a. | n.a. |
|  | PROSPER | 0.92 (0.63; 1.35) | 0.88 (0.60; 1.29) | 0.89 (0.60; 1.31) |
|  | LSADT | 1.15 (0.78; 1.71) | 1.18 (0.79; 1.76) | 1.15 (0.77; 1.71) |
|  | Leiden 85-plus | 1.08 (0.76; 1.55) | 1.13 (0.76; 1.67) | 1.11 (0.75; 1.65) |
|  | LLS F1 | 1.16 (0.86; 1.57) | n.a. | n.a. |
|  | Pooled estimate | 1.09 (0.91; 1.29) | 1.04 (0.83; 1.31) | 1.04 (0.83; 1.31) |
|  | | |  |  |
| IgG antibody quartile 4: | | |  |  |
|  | LLS F2^b^ | 0.94 (0.57; 1.54) | n.a. | n.a. |
|  | PROSPER | 0.88 (0.60; 1.29) | 0.83 (0.56; 1.23) | 0.82 (0.55; 1.22) |
|  | LSADT | 1.36 (0.91; 2.03) | 1.18 (0.78; 1.77) | 1.22 (0.82; 1.84) |
|  | Leiden 85-plus | 0.83 (0.56; 1.22) | 0.93 (0.62; 1.40) | 0.94 (0.62; 1.43) |
|  | LLS F1 | 1.28 (0.96; 1.71) | n.a. | n.a. |
|  | Pooled estimate | 1.06 (0.86; 1.30) | 0.96 (0.76; 1.22) | 0.98 (0.77; 1.24) |

HR: hazard ratio. CI: confidence interval. IgG: Immunoglobulin.

^a^ CMV seronegative individuals were the reference group.

^b^ For Leiden Longevity Study (LLS) F2, IgG antibody level was dichotomized instead of divided in quartiles due to rounded off values.

Cox regression analyses within individual cohorts were performed in 3 models:

Model 1: adjustment for age and sex (for PROSPER, also country and statin use).

Model 2: adjustment for model 1 plus Body Mass Index, education, smoking status, numbers of comorbidities and of medication.

Model 3: adjusted for model 2 plus log transformed C-reactive protein.
