# Supplementary material for: Associations of Cytomegalovirus Infection With All-Cause and Cardiovascular Mortality in Multiple Observational Cohort Studies of Older Adults
Source: J Infect Dis. 2020 Sep 10;223(2):238–46. doi: 10.1093/infdis/jiaa480 (PMC7857154; doi:10.1093/infdis/jiaa480)
Supplement: jiaa480_suppl_Supplementary_Table_S5 [file jiaa480_suppl_supplementary_table_s5.docx]

**Supplementary table S5: Associations between higher CMV IgG antibody quartiles with non-cardiovascular mortality, compared to the lowest CMV IgG quartile within individual cohorts.**

|  | Non-cardiovascular mortality, HR (95% CI) | | | |
| --- | --- | --- | --- | --- |
| *Cytomegalovirus* | Model 1 | | Model 2 | Model 3 |
| IgG antibody quartile 1^a^: 1 | | 1 | 1 | 1 |
| IgG antibody quartile 2: | | |  |  |
| LLS F2^b^ | n.a. | | n.a. | n.a |
| PROSPER | 0.87 (0.59; 1.29) | | 0.89 (0.60; 1.33) | 0.89 (0.60; 1.32) |
| LSADT | 1.04 (0.73; 1.50) | | 1.07 (0.74; 1.55) | 1.07 (0.74; 1.55) |
| Leiden 85-plus | 1.24 (0.90; 1.70) | | 1.27 (0.92; 1.75) | 1.29 (0.93; 1.78) |
| LLS F1 | 1.17 (0.82; 1.66) | | n.a. | n.a. |
| Pooled estimate | 1.09 (0.91; 1.30) | | 1.09 (0.89; 1.34) | 1.10 (0.89; 1.35) |
|  |  | |  |  |
| IgG antibody quartile 3: | | |  |  |
| LLS F2^b^ | n.a | | n.a. | n.a |
| PROSPER | 0.86 (0.57; 1.28) | | 0.85 (0.56; 1.27) | 0.84 (0.56; 1.27) |
| LSADT | 0.91 (0.62; 1.34) | | 0.94 (0.63; 1.39) | 0.94 (0.64; 1.39) |
| Leiden 85-plus | 1.03 (0.76; 1.40) | | 1.05 (0.76; 1.45) | 1.05 (0.76; 1.45) |
| LLS F1 | 1.11 (0.77; 1.60) | | n.a. | n.a. |
| Pooled estimate | 0.99 (0.82; 1.18) | | 0.96 (0.78; 1.19) | 0.96 (0.78; 1.19) |
|  |  | |  |  |
| IgG antibody quartile 4: | | |  |  |
| LLS F2^b^ | 1.07 (0.59; 1.94) | | n.a. | n.a |
| PROSPER | 0.79 (0.52; 1.20) | | 0.80 (0.52; 1.21) | 0.78 (0.52; 1.19) |
| LSADT | 1.06 (0.71; 1.58) | | 0.95 (0.64; 1.42) | 0.96 (0.64; 1.44) |
| Leiden 85-plus | 0.80 (0.58; 1.10) | | 0.85 (0.62; 1.18) | 0.89 (0.64; 1.24) |
| LLS F1 | 1.24 (0.86; 1.78) | | n.a. | n.a. |
| Pooled estimate | 0.96 (0.80; 1,15) | | 0.86 (0.70; 1.07) | 0.88 (0.71; 1.09) |

HR: hazard ratio. CI: confidence interval. IgG: Immunoglobulin.

^a^ CMV IgG antibody quartile 1 was the reference group.

^b^ For Leiden Longevity Study (LLS) F2, IgG antibody level was dichotomized instead of divided in quartiles due to rounded off values.

Cox regression analyses within individual cohorts were performed in 3 models:

Model 1: adjustment for age and sex (for PROSPER, also country and statin use).

Model 2: adjustment for model 1 plus Body Mass Index, education, smoking status, numbers of comorbidities and of medication.

Model 3: adjusted for model 2 plus log transformed C-reactive protein.
