# Supplementary figures and images for: Associations of Cytomegalovirus Infection With All-Cause and Cardiovascular Mortality in Multiple Observational Cohort Studies of Older Adults
Source: J Infect Dis. 2020 Sep 10;223(2):238–46. doi: 10.1093/infdis/jiaa480 (PMC7857154; doi:10.1093/infdis/jiaa480)

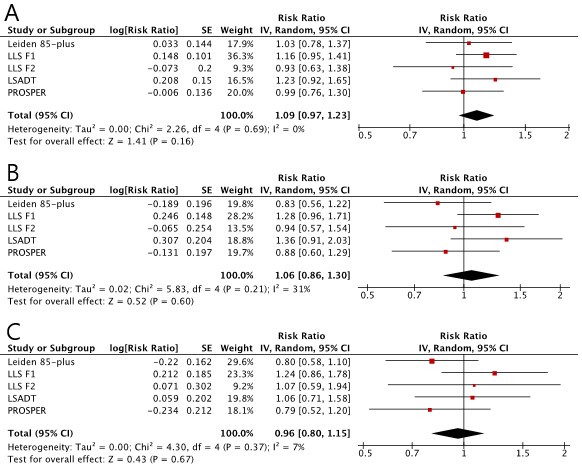

Supplement: jiaa480_suppl_Supplementary_Figure_1 [file jiaa480_suppl_supplementary_figure_1.jpeg]
